# Transmission of Severe Fever with Thrombocytopenia Syndrome Virus to Human from Nonindigenous Tick Host, Japan

**DOI:** 10.3201/eid3011.240912

**Published:** 2024-11

**Authors:** Qiang Xu, Takeshi Nabeshima, Koichiro Hamada, Takashi Sugimoto, Mya Myat Ngwe Tun, Kouichi Morita, Hirotomo Yamanashi, Takahiro Maeda, Koya Ariyoshi, Yuki Takamatsu

**Affiliations:** Nagasaki University, Nagasaki, Japan (Q. Xu, T. Nabeshima, K. Hamada, T. Sugimoto, M.M. Ngwe Tun, K. Morita, H. Yamanashi, T. Maeda, K. Ariyoshi, Y. Takamatsu); Shimane University, Izumo, Japan (M.M. Ngwe Tun)

**Keywords:** severe fever with thrombocytopenia syndrome, severe fever with thrombocytopenia syndrome virus, ticks, viruses, vector-borne infections, tickborne infections, febrile diseases, Japan

## Abstract

We report a human case of severe fever with thrombocytopenia syndrome virus infection transmitted by a tick, confirmed by viral identification. *Haemaphysalis aborensis*, a tick species not native to Japan that has been observed to transmit the virus to humans, is now recognized as a potential vector of this virus in Japan.

Blood-feeding ticks can transmit viruses to vertebrates, including humans. A previously unknown flavivirus, Saruyama virus, was detected in Japan in 2018 (*1*); similar viral sequences have also been identified in wild deer and boars in Japan. Severe fever with thrombocytopenia syndrome (SFTS) is an emerging tickborne disease caused by SFTS virus (SFTSV), which belongs to the family Phenuiviridae, genus *Bandavirus*. The first SFTS case was reported in China in 2010 (*2*), followed by cases in Japan and South Korea in 2013 (*3*,*4*); those 3 countries are the primary endemic areas for SFTSV. 

Tick bites are the primary route of SFTSV transmission (*2*,*5*). *Haemaphysalis longicornis* ticks, native to east Asia, have been identified as a major SFTSV vector (*2*,*6*). SFTSV cases have been reported in several countries in Southeast Asia, including Vietnam and Thailand, and in South Asia, including Pakistan (*7*), suggesting that SFTSV might expand from endemic regions in tandem with its host animals or through tick migration. In Japan, several tick species, including *H.* *flava*, *H. megaspinosa*, *H. kitaokai*, *H. formosensis*, and *H. hystricis*, carry the SFTSV genome (*8*,*9*). The mortality rate for SFTS infection ranges from 5% to 28%; the elderly are at higher risk for fatal clinical outcomes (*10*), indicating its potential public health consequences. No antiviral drugs or vaccines are available for SFTSV infection. 

We report a human case of SFTS transmitted by a novel tick host, *H. aborensis*, a tick not endemic to Japan that has been identified as a potential vector of SFTSV, indicating possible expansion of habitats of infectious ticks. That finding highlights the importance of comprehensive viral genome analysis as part of routine tick-borne viral surveillance. We obtained informed consent for publication from the patient and ethics approval from the Clinical Research Ethics Committee of Nagasaki University Hospital (record no. 23112012). 

## The Study 

An 80-year-old female patient with a medical history of hypertension, bronchial asthma, and cerebral aneurysm (postoperative), but no history of smoking, alcohol consumption, or recent travel, experienced fever and dizziness. The case-patient resided in Nagasaki, Japan, close to the forest, and reported that she frequently encountered wild animals, such as wild boars and civets. She engaged in daily activities, regularly tended her garden, and had no companion animals. She sought medical consultation with a primary care physician on day 3 after onset of symptoms. 

**Figure 1:**
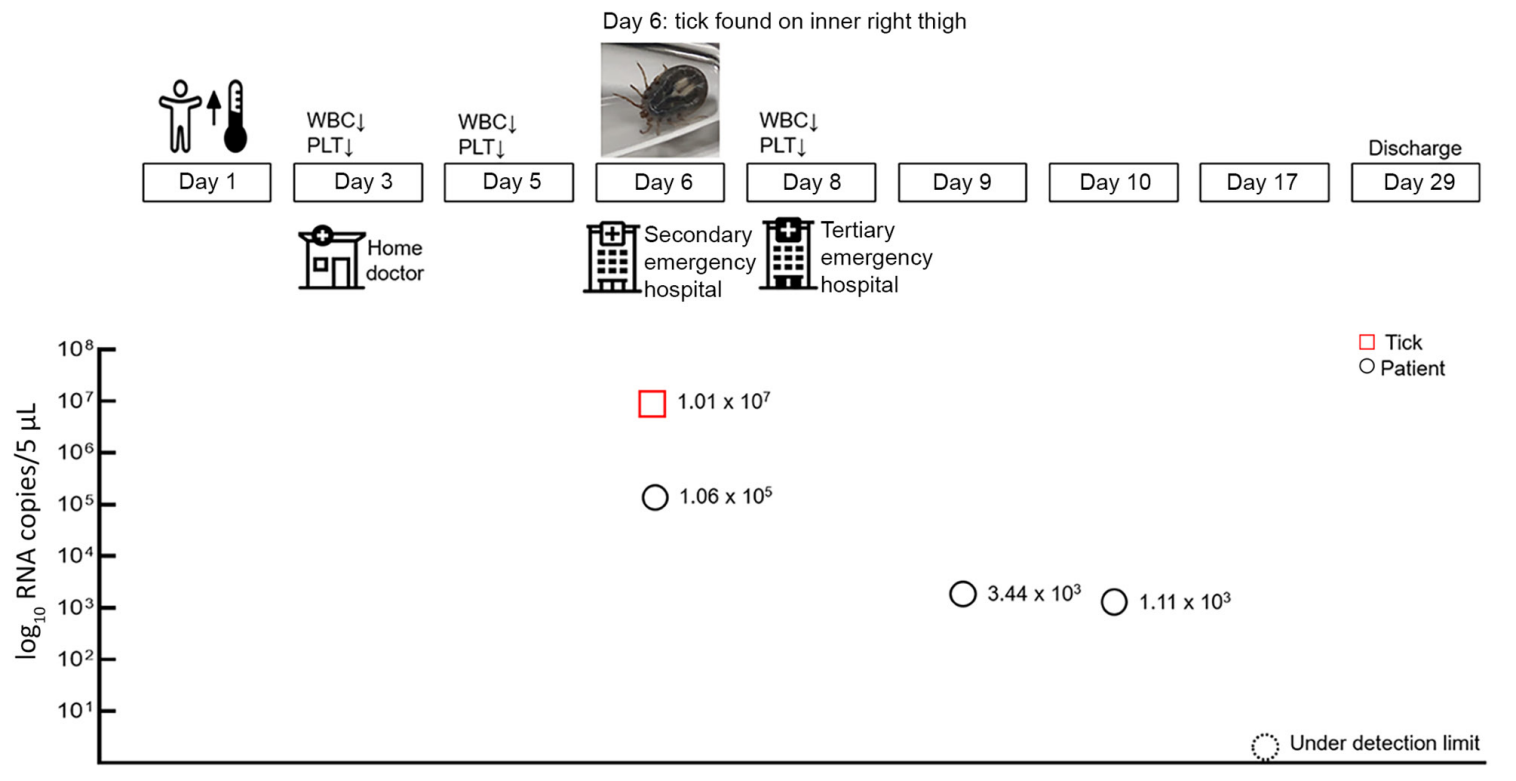
Timeline of SFTSV progression in a human patient in Japan and photograph of *Haemaphysalis aborensis* tick collected from the patient. WBC, white blood cells (leukocytes); PLT, platelets; SFTSV, severe fever with thrombocytopenia syndrome virus.

Blood tests revealed a drop in her leukocyte count to 3,180 cells/μL (reference range 3,300–8,600 cells/μL) and platelet count to 104,000/μL (reference range 158,000–348,000/μL). Subsequent blood tests showed further decreases in leukocytes to 1,690 cells/μL and platelets to 72,000/μL. On day 5, the case-patient was referred to a secondary emergency hospital for further evaluation. A blood-engorged tick was found on her inner right thigh (Figure 1). Presence of leukopenia, thrombocytopenia, and tick bites indicated SFTSV infection. A serum specimen from the patient was sent to the laboratory at Nagasaki City Health Center, which is responsible for administrative inspections for SFTS diagnosis. The results revealed SFTSV positivity on day 12 after symptom onset. 

The patient was transferred from the secondary hospital to the Department of Internal Medicine of Infectious Diseases at Nagasaki University Hospital, a tertiary emergency hospital, on day 8 after onset. Physical examination at time of admission indicated a body temperature of 36.0°C, heartbeat of 66 beats/min, blood pressure of 121/72 mm Hg, SpO_2_ of 94% (room air), and respiratory rate of 28 breaths/min. The patient’s level of consciousness was unclear, but she responded when called, which is indicative of a II-10 rating on the Japan Coma Scale. Blood chemical examination demonstrated results with reference ranges for hemoglobin (12.3 g/dL), sodium (137 mEq/L), potassium (3.8 mEq/L), chloride (108 mEq/L), blood urea nitrogen (6 mg/dL), creatinine (0.7 mg/dL), and C-reactive protein (0.04 mg/dL). Compared with earlier test results, we noted further decreases in leukocyte count, to 2,300 cells/μL (neutrophils 920 cells/μL, lymphocytes 1,080 cells/μL), and platelet count, to 53,000/μL; we also saw increases in aspartate transferase (133 U/L, reference range 13–30 U/L), alanine transaminase (64 U/L, reference range 7–23 U/L), lactate dehydrogenase (770U/L, reference range 124–222 U/L), and creatine kinase (471 U/L, reference range 41–153 U/L). Urine examination revealed high protein 2+ and occult blood 2+ results. Results of blood cultures on days 6 and 10 and urine cultures on day 10 after onset were negative for bacterial infections. The patient gradually recovered and was discharged on day 29 after symptom onset without any specified lasting effects. 

We sent the tick from the patient and serum specimens collected on days 6, 9, 10, and 17 after onset to the Department of Virology, Institute of Tropical Medicine, at Nagasaki University for examination. We extracted total RNA from the homogenized tick sample (Appendix) and subjected serum specimens to quantitative reverse-transcription PCR (qRT-PCR) (Appendix). The specimen from day 6 demonstrated the highest number of SFTSV RNA copies (1.06 × 10^5^/5 μL). The SFTSV RNA copies in the serum specimens decreased and were undetectable on day 17 after onset (Figure 1). Homogenates from the tick demonstrated a substantially higher number of SFTSV RNA copies (1.01 × 10^7^/5 μL) than the patient samples. We isolated viruses only from the tick, not from patient specimens. 

**Figure 2:**
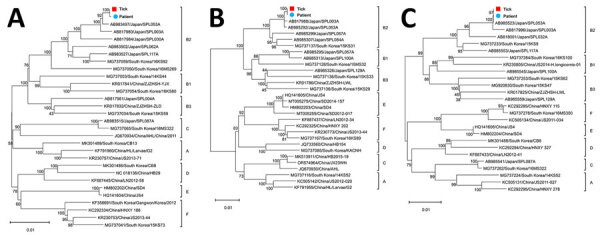
Phylogenetic trees based on the coding sequence of the SFTSV segments from a human patient in Japan and a *Haemaphysalis aborensis* tick collected from the patient. A) Large segment; B) medium segment; C) small segment. Blue circles indicate patient-derived SFTSV strains and red squares tick-derived strains from this study. Labels at right of each tree represent SFTSV genotypes A‒F. We derived the phylogenetic trees using the maximum-likelihood method and general time-reversible model and ran 1,000 bootstrap replicates using MEGA 11.0.13 (https://www.megasoftware.net). Scale bars indicate the number of base differences per site. SFTSV, severe fever with thrombocytopenia syndrome virus.

To explore the genomic similarity of SFTSV strains derived from tick and human samples, we determined the full-length protein-coding sequences of the large (L), medium (M), and small (S) segments of viruses from the tick (GenBank accession nos. PP813867–9) and patient (accession nos. PP839300–2) by using next-generation sequencing (Appendix). We conducted phylogenetic analysis using MEGA11 (https://www.megasoftware.net) to determine the genetic relationships between the sequences from our study and previously identified SFTSV sequences from countries in Asia, including Japan (*11*). The sequences of patient- and tick-derived SFTS L/M/S segments were identical. SFTSV identified in our study’s belonged to B-2 clade (Figure 2, panels A–C), the genotype most prevalent in Japan and South Korea (*10*). 

**Figure 3:**
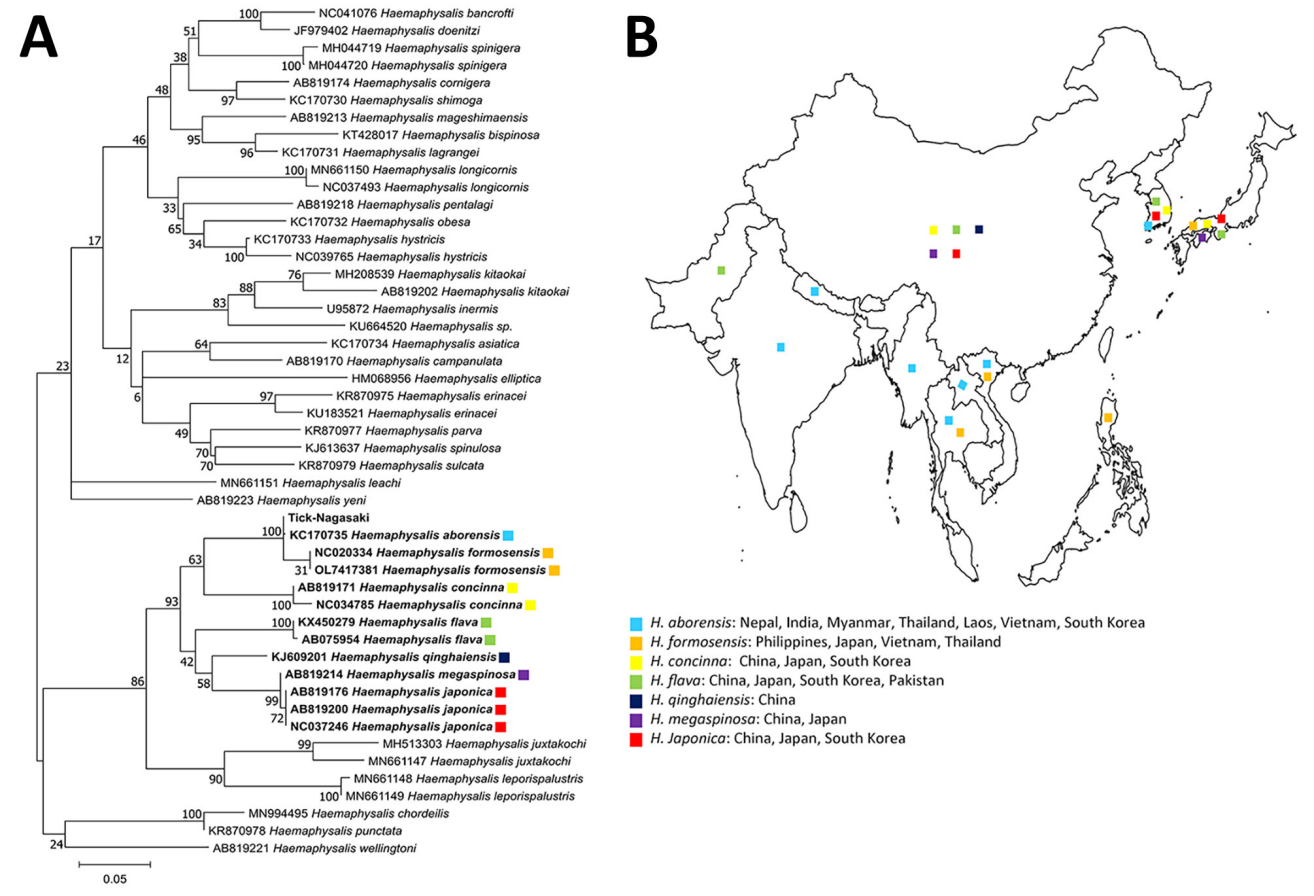
Phylogenetic tree (A) and geographic distribution (B) of 36 tick species from the genus *Haemaphysalis*. Bold indicates tick sequences analyzed in this study; Tick-Nagasaki indicates tick collected from a human patient in Japan who had severe fever with thrombocytopenia syndrome virus. Colors indicate locations where ticks have been found. We used 49 16S rRNA sequences to construct the maximum-likelihood tree based on 1,000 replicates in MEGA 11.0.13 (https://www.megasoftware.net). Bootstrap values are indicated next to the branches. Scale bar indicates nucleotide substitutions per site.

Morphologic characteristics (Figure 1) identified the tick collected from the patient as belonging to the genus *Haemaphysalis*. To confirm species identification, we sequenced the 16S ribosomal RNA (accession no. PP813416) (Appendix). Phylogenetic analysis identified it as most closely related to *H. aborensis*, a species not endemic to Japan (Figure 3, panel A). We found no previous reports of SFTSV isolation or gene detection in *H. aborensis* ticks. 

*H. aborensis* ticks are primarily distributed in Nepal and India in South Asia and Laos, Vietnam, and Thailand in Southeast Asia (Figure 3, panel B) (*12*); porcupines, wild boars, and deer are the primary hosts (*12*). A previous study identified *H. aborensis* ticks collected from *Turdus pallidus* (pale thrush) on Hong Island, South Korea (*13*). The *T. pallidus* thrush is a migratory bird that breeds in areas from northeast China to far eastern Russia and overwinters in southern and central Japan, South Korea, and southern China (*14*). Because the B-2 clade has been isolated only in Japan and South Korea (*10*), SFTSV-infected ticks were likely carried by infected birds from South Korea. Although it is possible that ticks were carried by birds from South Korea, then acquired and transmitted SFTSV through infected animals in Japan, this scenario is unlikely because *H. aborensis* ticks had not been previously identified in Japan. 

## Conclusions 

We report a case of tick-transmitted SFTSV infection in a human patient. Virus isolation and identification of the tick species confirmed that *H. aborensis* ticks can transmit SFTSV to humans. The phylogenetic analysis revealed no differences between sequences of SFTSV from the tick and the patient. Identifying an additional host tick highlights the importance of routine tick surveillance for monitoring SFTSV expansion. 

Appendix Additional information from study of human case of severe fever thrombocytopenia syndrome, Japan.
