# Supplementary material for: Transmission of Severe Fever with Thrombocytopenia Syndrome Virus to Human from Nonindigenous Tick Host, Japan
Source: Emerg Infect Dis. 2024 Nov;30(11):2419–23. doi: 10.3201/eid3011.240912 (PMC11521155; doi:10.3201/eid3011.240912)
Supplement: Appendix — Additional information from study of human case of severe fever thrombocytopenia syndrome, Japan. [file 24-0912-Techapp-s1.pdf]

Article DOI: https://doi.org/10.3201/eid3011.240912

EID cannot ensure accessibility for supplementary materials supplied by authors. Readers who have difficulty accessing supplementary content should contact the authors for assistance.

Transmission of Severe Fever with Thrombocytopenia Syndrome Virus to Human from Nonindigenous Tick Host, Japan

Appendix

Additional Methods

Tick Homogenization and Extract Total RNA

The tick collected from the patient was homogenized following procedures from a previous study (*1*). Briefly, tick samples were homogenized in 500 µL of phosphate-buffered saline (PBS) containing 2% fetal bovine serum (FBS) with P/S (Biowest, https://biowest.net), and 100 IU/mL penicillin and 100 µg/mL streptomycin (Sigma-Aldrich, https://www.sigmaaldrich.com), using the Bead Crusher µT-12 (TAITEC Corporation, https://e-taitec.com) and 2 mm stainless beads. RNA was extracted using ISOGEN-II (Nippon Gene, https://nippongene.com/index.html), following manufacturer instructions.

RT-qPCR Reaction

The quantitative reverse-transcription PCR (RT-qPCR) for SFTSV RNA detection was described previously (*2*). Briefly, total RNA was extracted from the patient's serum by ISOGEN-LS (Nippon Gene). The total RNA from the patient’s serum and tick homogenate was subjected to RT-qPCR using the One-Step PrimeScript RT-PCR Kit (Takara Bio,

https://www.takarabio.com) with a specific primer set and probe (Appendix Table), on the 7500 real-time RT-PCR System (Applied Biosystems, https://www.thermofisher.com).

Virus Isolation

Virus isolation procedures followed a previous study (*3*). Briefly, patient serum specimens collected on day 6 after onset and tick lysate were filtered through a Millipore 0.22 µm column (https://www.sigmaaldrich.com), and 50 µL of each was inoculated into T25 flasks containing Vero E6 African green monkey kidney cells. Next, 500 µL of Dulbecco modified Eagle medium (DMEM; Fujifilm Wako Pure Chemical, https://www.fujifilm.com) without FBS was added, and the cells were incubated at 37°C for 1 hour. Subsequently, 7 mL of DMEM with P/S and 2% FBS were added, and the cells were incubated at 37°C under 5% CO_2 for 7 days. On day 7 after inoculation, the cell supernatant was collected and 500 µL inoculated into fresh Vero E6 cells in T75 flasks for a secondary passage. Each passage was confirmed by observing the cytopathic effect and RT-qPCR.

Sequencing of SFTSV Full-Length Coding Region and Tick 16S rRNA

The RNA was extracted from tick lysate to achieve the complementary DNA synthesis by using ReverTra Ace-α-TM (Toyobo, https://www.toyobo-global.com) with random primers (Takara Bio). The 16S ribosomal RNA (rRNA) gene was amplified according to a previous study (*4*) (Appendix Table) and cloned into the Topo vector using the Zero Blunt TOPO PCR Cloning Kit (ThermoFisher Scientific, https://www.thermofisher.com). Subsequently, sequencing was performed with specific primers (Appendix Table) on an ABI 3500 Genetic Analyzer (Applied Biosystems).

RNA was extracted for next-generation sequencing from the patient's serum specimens from day 6 after onset and the tick lysate. First, a whole transcriptome library was constructed using the Ion Total RNA-Seq Kit v2 (ThermoFisher Scientific) and sequenced on the Ion Proton semiconductor sequencer (ThermoFisher Scientific). The raw sequencing data was processed

using FASTX-Toolkit (*5*) and de novo transcriptome assembly using Trinity RNA-Seq (*6*). The sequences were aligned with the ref-Seq from the National Center for Biotechnology Information (NCBI) to obtain the SFTSV sequencing results.

Appendix Table. The details of the primers used in this study include the following information for each primer: gene region, and nucleotide sequence of the primers and probes.

Primer	Region of gene	Sequence
RT-qPCR		
SFTSV-S-qPCR-F	S segment	5'- TGTCAGAGTGGTCCAGGATT-3'
SFTSV-S-qPCR-R		5'- ACCTGTCTCCTTCAGCTTCT-3'
SFTSV-S-qPCR probe		FAM-TGGAGTTTGGTGAGCAGCAGC-BHQ1
Tick identification		
16S-F	16S rRNA	5'-TTAAATTGCTGTRGTATT-3'
16S-R		5'-CCGGTCTGAACTCASAWC-3'
M13-F	Tick_16S rRNA-Topo	5'-GTAAAACGACGGCCAG-3'
M13-R	clone	5'-CAGGAAACAGCTATGAC-3'

References

1. Kim UJ, Kim DM, Kim SE, Kang SJ, Jang HC, Park KH, et al. Case report: detection of the identical virus in a patient presenting with severe fever with thrombocytopenia syndrome encephalopathy and the tick that bit her. BMC Infect Dis. 2018;18:181. PubMed https://doi.org/10.1186/s12879-018-3092-y

2. Yun SM, Park SJ, Kim YI, Park SW, Yu MA, Kwon HI, et al. Genetic and pathogenic diversity of severe fever with thrombocytopenia syndrome virus (SFTSV) in South Korea. JCI Insight. 2020;5:e129531. PubMed https://doi.org/10.1172/jci.insight.129531

3. Takahashi T, Maeda K, Suzuki T, Ishido A, Shigeoka T, Tominaga T, et al. The first identification and retrospective study of Severe Fever with Thrombocytopenia Syndrome in Japan. J Infect Dis. 2014;209:816–27. PubMed https://doi.org/10.1093/infdis/jit603

4. Thompson AT, Dominguez K, Cleveland CA, Dergousoff SJ, Doi K, Falco RC, et al. Molecular characterization of *Haemaphysalis* species and a molecular genetic key for the identification of *Haemaphysalis* of North America. Front Vet Sci. 2020;7:141. PubMed https://doi.org/10.3389/fvets.2020.00141

5. FASTX-Toolkit [cited 2024 May 7]. http://hannonlab.cshl.edu/fastx_toolkit/

6. Grabherr MG, Haas BJ, Yassour M, Levin JZ, Thompson DA, Amit I, et al. Full-length transcriptome assembly from RNA-Seq data without a reference genome. Nat Biotechnol. 2011;29:644–52. PubMed https://doi.org/10.1038/nbt.1883
